# Intention to Use Large Language Models Among Clinical Nurses in China With Prior Familiarity With or Experience Using LLMs: A Qualitative Study

**DOI:** 10.1155/jonm/7972792

**Published:** 2026-08-29

**Authors:** Xu Li, Xu Hu, Huiting Xu, Jingjing Guo, Hailing Ju, Pin Yu

**Affiliations:** ^1^ Department of Central ICU, The First Affiliated Hospital of Soochow University, Suzhou, China, sdfyy.cn; ^2^ School of Medicine, Tongji University, Shanghai, China, tongji.edu.cn; ^3^ Department of Operating Room, Qingpu Branch of Zhongshan Hospital Affiliated to Fudan University, Shanghai, China, fudan.edu.cn; ^4^ Department of Nursing, Shanghai Tenth People’s Hospital, Shanghai, China, shdsyy.com.cn

**Keywords:** clinical nurses, intention to use, large language models, qualitative research, UTAUT

## Abstract

**Objective:**

To explore the intention to use large language models (LLMs) among clinical nurses with prior familiarity with or experience using LLMs, and to examine how relevant facilitators, constraints, perceived risks, and professional boundary considerations were reflected in such intention.

**Methods:**

Guided by the Unified Theory of Acceptance and Use of Technology (UTAUT), this descriptive qualitative study employed semistructured interviews with 17 clinical nurses from different hospital levels, specialties, and roles. Data were analyzed using directed content analysis.

**Results:**

Six themes were identified: performance expectancy, effort expectancy, social influence, facilitating conditions, perceived risk, and professional boundaries and identity. Participants expressed a positive but conditional intention to use LLMs. Performance expectancy, effort expectancy, social influence, and facilitating conditions were reflected in participants’ accounts as perceived enabling conditions, whereas perceived risk and professional boundaries and identity were described as important sources of caution. Participants acknowledged the potential supportive value of LLMs in standardized and relatively low‐risk tasks, such as documentation, knowledge support, and teaching‐related work, but emphasized that LLMs should not replace nurses’ clinical judgment, accountability, or humanistic communication.

**Conclusion:**

Among clinical nurses with prior familiarity with or experience using LLMs, intention to use these tools was reflected in participants’ accounts of perceived value, usability, organizational conditions, risk appraisal, and professional boundaries. Conditional intention should be understood as an interpretive finding rather than as a validated new construct or a formal extension of UTAUT. These findings provide context‐specific insights for cautious pilot testing, governance, and implementation of LLM‐supported nursing applications.

**Implications for Nursing Management:**

Nurse managers may consider cautious pilot testing of LLM‐supported nursing applications in low‐risk and standardized tasks. Priorities include defining appropriate‐use boundaries, data security, and accountability requirements, ensuring human review, providing tiered training, and evaluating usability, workflow fit, safety concerns, and nurses’ feedback before wider implementation.

## 1. Introduction

The global imbalance between the supply of and demand for nursing staff has become increasingly prominent, while nurses’ workload and occupational stress continue to rise, posing a major systemic challenge to healthcare quality and patient safety. According to the World Health Organization, the global nursing workforce is projected to face a shortage of 10.6 million nurses by 2030, and approximately 4.7 million nurses are expected to retire over the next decade [1]. Population aging, the growing burden of chronic diseases, expanding healthcare needs, workforce aging, and insufficient educational resources have jointly contributed to widening nursing shortages, persistent understaffing, and increased overtime, which in turn may lead to burnout, stronger turnover intention, and declines in the quality of care [2]. Previous studies have shown that nursing shortages and the resulting increase in workload are important external factors affecting continuity of care and patient outcomes [3]. In this context, health information technology and digital tools have been regarded as potential means of alleviating nursing workforce pressure, as they may improve nursing efficiency and release more time for direct patient care through workflow redesign, documentation automation, mobile ward rounds, and clinical decision support [4].

Against this background, large language models (LLMs), represented by tools such as ChatGPT and DeepSeek, have attracted increasing attention in healthcare because of their strengths in natural language understanding, text generation, knowledge integration, and intelligent question answering [5]. Emerging evidence suggests that LLMs have shown considerable potential in medical education, health consultation, research assistance, medical documentation, and clinical decision support [6–8]. Compared with traditional information systems, LLMs offer greater interactivity, learning capability, and generative capacity, enabling them to support nurses in information retrieval, language organization, and knowledge synthesis. Their application provides a new technological pathway for advancing nursing from informatization to intelligent care, with the potential to reduce workload, improve efficiency, and optimize the quality of nursing care in areas such as nursing documentation, health education, teaching and training, research support, and nursing management [9–11]. However, these potential benefits should be understood cautiously, because the actual value and safety of LLMs in nursing depend on their reliability, workflow fit, governance arrangements, and users’ ability to critically evaluate AI‐generated outputs.

Nurses are core practitioners within the healthcare system and are also the direct users and key stakeholders in the implementation of LLMs in clinical nursing settings. Nurses’ knowledge, skills, and critical judgment may influence the safe integration and perceived practical value of LLMs in nursing practice. However, nurses currently face multidimensional barriers when engaging with generative AI tools such as LLMs. For example, Pare et al. reported that nurses have limited understanding of the basic principles, scope of application, and potential limitations of AI and that the lack of targeted AI literacy training reduces their ability to evaluate both the functions and risks of these tools; “lack of knowledge/familiarity” has been identified as one of the main barriers to clinical nurses’ adoption of AI tools [12]. Nurses also express concerns about the interpretability and reliability of AI‐generated outputs, worrying that AI errors, data privacy breaches, and unclear ethical accountability may adversely affect patient safety. High perceived risk may weaken their intention to use AI and increase resistance to change [13]. In addition, some nurses are concerned that AI may replace parts of their professional role or weaken the humanistic care dimension of nursing. Such feelings of being “replaced” may further reduce support for AI and willingness to try it, particularly in settings characterized by job insecurity or insufficient institutional support [14]. Moreover, studies have shown that if AI tools cannot be effectively integrated into existing nursing workflows, information systems, and institutional arrangements, they may instead increase work complexity, time costs, and additional burden. At the same time, insufficient facilitating conditions (FCs) at the organizational level are also important external barriers to nurses’ adoption of AI [15].

Current research on the application of LLMs in nursing has been conducted predominantly using quantitative approaches, mainly focusing on levels of technology acceptance, patterns of use, influencing factors, and intervention effects [16–19]. Such studies are helpful for providing an overall understanding of nurses’ attitudes toward new technologies; however, they are less able to capture nurses’ specific views in real‐world practice settings and the underlying reasons for those views. Although a small number of studies have adopted qualitative methods, their participants have largely been students, with primary attention given to cognitive status, educational support, learning experiences, and academic applications [20–22]. Compared with students, clinical nurses must judge the usability and safety of LLMs within work contexts characterized by high risk, high responsibility, and strong professional boundaries. As a result, the logic underlying their technology adoption is more complex and of greater practical relevance.

A recent Chinese qualitative preprint has explored clinical nurses’ willingness to use LLMs and their application needs, reporting themes related to cognition and acceptance, application scenario requirements, learning and training needs, and concerns or barriers associated with LLM use [23]. This study provides useful preliminary descriptive evidence regarding Chinese clinical nurses’ views and practical needs. However, as a preprint, its findings should be interpreted cautiously. In addition, its primary focus was on willingness and application demands, whereas further theoretically informed research is needed to examine how facilitators, constraints, perceived risks, and professional boundary considerations are reflected in nurses’ intention to use LLMs.

Although several issues identified in this study, such as usefulness, usability, training needs, privacy concerns, organizational support, and professional autonomy, have been reported in previous AI or LLM‐related nursing studies, our contribution lies in using UTAUT to interpret how these issues are reflected in nurses’ intention to use LLMs. In addition, perceived risk and professional boundaries were used as sensitizing concepts to highlight nursing‐specific concerns related to safety, accountability, human judgment, and the limits of AI‐supported practice.

Therefore, the objective of this study was to explore the intention to use LLMs among clinical nurses with prior familiarity with or experience using LLMs and to examine how established considerations such as perceived usefulness, usability, organizational conditions, risk appraisal, and professional boundaries were reflected in such intention. Guided by UTAUT, this study used the four UTAUT constructs as predefined analytical categories and treated perceived risk and professional boundaries as sensitizing concepts included in the interview guide. Rather than proposing a new construct or a formal extension of UTAUT, this study aimed to provide an interpretive account of nurses’ conditional intention to use LLMs in real‐world nursing practice. The study was guided by the following research question: How do clinical nurses with prior familiarity with or experience using LLMs perceive these tools, and what factors are reflected in their intention to use LLMs in nursing practice?

## 2. Participants and Methods

### 2.1. Participants

This study adopted a descriptive qualitative design. From January to March 2026, nurses were recruited from seven hospitals in Jiangsu and Shanghai using purposive sampling with a maximum‐variation strategy. To obtain rich and diverse data, participants were selected from different hospital levels, departments, and positions, including frontline clinical nurses, clinical preceptors, head nurses, and nursing administrators. A total of 17 nurses were approached and invited to participate in the study; all agreed to participate, and none declined or withdrew.

The inclusion criteria were as follows: (1) holding a valid nursing license and having at least 1 year of nursing‐related work experience; (2) having some knowledge of or experience using LLMs; (3) providing informed consent and voluntarily agreeing to participate in the study; and (4) being able to express personal views clearly. The exclusion criteria were as follows: (1) withdrawal during the interview process and (2) incomplete interview data. The sample size was determined according to the principle of data saturation; that is, no new themes or categories emerged during data analysis [24].

To protect participants’ privacy, their real names were removed and replaced with codes (P1, P2, P3, etc.). Details are shown in Table 1.

**TABLE 1:** General characteristics of the participants.

Participant no.	Gender	Age	Educational level	Hospital type	Department	Years of work experience	Professional title	Position	Nursing hierarchy level	Main LLMs used	Main use context	Basis of views	Frequency of use
P1	Female	45	Bachelor’s degree	Tertiary hospital	Intensive care unit	20	Associate chief nurse	Head nurse	G	Doubao; DeepSeek	Clinical supportive tasks; knowledge support/work preparation; teaching/training; research/academic writing; administrative/management tasks	Mixed clinical and nonclinical professional use	Regular use
P2	Male	36	Master’s degree	Tertiary hospital	General surgery	12	Nurse in charge	Head nurse	G	Doubao; DeepSeek; ChatGPT	Clinical supportive tasks; knowledge support/work preparation; teaching/training; research/academic writing; administrative/management tasks	Mixed clinical and nonclinical professional use	Regular use
P3	Female	27	Master’s degree	Tertiary hospital	Intensive care unit	2	Nurse	None	N1	Doubao; DeepSeek; ChatGPT	Clinical supportive tasks; knowledge support/work preparation; research/academic writing	Mixed clinical and nonclinical professional use	Sustained use
P4	Male	28	Bachelor’s degree, currently pursuing a master’s degree	Tertiary hospital	Intensive care unit	7	Senior nurse	None	N2	Doubao; ChatGPT	Clinical supportive tasks; knowledge support/work preparation; teaching/training; research/academic writing; administrative/management tasks	Mixed clinical and nonclinical professional use	Sustained use
P5	Female	29	Bachelor’s degree, currently pursuing a master’s degree	Tertiary hospital	Operating room	8	Nurse in charge	None	N2	Doubao; DeepSeek; ChatGPT; Gemini	Knowledge support/work preparation; teaching/training; research/academic writing	Mixed clinical and nonclinical professional use	Sustained use
P6	Male	25	Associate degree	Secondary hospital	Intensive care unit	5	Senior nurse	None	N1	Doubao	Knowledge support/work preparation	Mixed clinical and nonclinical professional use	Regular use
P7	Female	26	Associate degree	Tertiary hospital	Intensive care unit	6	Senior nurse	None	N2	Doubao	Clinical supportive tasks; knowledge support/work preparation	Mixed clinical and nonclinical professional use	Regular use
P8	Female	37	Bachelor’s degree	Tertiary hospital	Intensive care unit	17	Nurse in charge	None	N3	Doubao; Qwen	Clinical supportive tasks; knowledge support/work preparation; administrative/management tasks	Mixed clinical and nonclinical professional use	Regular use
P9	Female	35	Doctoral degree	Tertiary hospital	Nursing department	10	Associate chief nurse	Secretary	N3	ChatGPT; Gemini	Clinical supportive tasks; knowledge support/work preparation; teaching/training; research/academic writing; administrative/management tasks	Mixed clinical and nonclinical professional use	Sustained use
P10	Male	25	Bachelor’s degree	Tertiary hospital	Infection department	2	Nurse	None	N1	Doubao; DeepSeek	Knowledge support/work preparation	Direct clinical work‐related use	Regular use
P11	Male	36	Bachelor’s degree	Tertiary hospital	Intensive care unit	15	Associate chief nurse	Clinical preceptor	N3	Doubao; Qwen; ERNIE Bot	Clinical supportive tasks; knowledge support/work preparation; teaching/training; administrative/management tasks	Mixed clinical and nonclinical professional use	Regular use
P12	Male	29	Bachelor’s degree	Tertiary hospital	Psychiatry	8	Nurse in charge	None	N2	Doubao; Qwen; ERNIE Bot	Knowledge support; teaching/training	Nonclinical professional use	Regular use
P13	Female	33	Associate degree	Secondary hospital	Neurology	14	Nurse in charge	None	N3	Doubao; DeepSeek	Knowledge support/work preparation; teaching/training	Direct clinical work‐related use	Regular use
P14	Male	29	Master’s degree	Tertiary hospital	Emergency department	5	Nurse in charge	None	N2	Doubao; DeepSeek	Clinical supportive tasks; knowledge support/work preparation	Mixed clinical and nonclinical professional use	Sustained use
P15	Female	30	Associate degree	Tertiary hospital	Infectious diseases department	9	Nurse in charge	None	N3	Doubao; DeepSeek; Qwen	Knowledge support/work preparation; administrative/management tasks	Direct clinical work‐related use	Regular use
P16	Male	29	Bachelor’s degree	Tertiary hospital	Pediatrics	8	Senior nurse	None	N2	Doubao; DeepSeek	Clinical supportive tasks; knowledge support/work preparation	Mixed clinical and nonclinical professional use	Sustained use
P17	Female	36	Bachelor’s degree	Tertiary hospital	Pediatrics	15	Associate chief nurse	Clinical preceptor	N3	Doubao; DeepSeek	Knowledge support/work preparation; teaching/training; administrative/management tasks	Mixed clinical and nonclinical professional use	Regular use

*Note:* In the nursing hierarchy column, N1 refers to developing nurses, N2 to proficient nurses, N3 to expert nurses, and G to nursing managers. Frequency of use was classified based on participants’ self‐described prior or current use of LLMs during the interviews, rather than by a standardized quantitative frequency measure. “Regular use” refers to repeated use of LLMs for specific professional or learning‐related tasks, but without clear evidence that the tool had been continuously integrated into routine work or study. “Sustained use” refers to continued or relatively stable use over time, especially when participants described using LLMs across multiple recurring professional, learning, teaching, research, or administrative tasks. These categories should, therefore, be interpreted as descriptive classifications of participants’ self‐reported usage experience, rather than as quantitatively measured levels of usage frequency.

### 2.2. Methods

#### 2.2.1. Theoretical Framework

This study was guided by the Unified Theory of Acceptance and Use of Technology (UTAUT) [25]. The UTAUT model includes four core constructs—performance expectancy (PE), effort expectancy (EE), social influence (SI), and FC—with behavioral intention and use behavior as outcome variables, while gender, age, experience, and voluntariness of use are treated as moderating variables. According to this theory, PE, EE, and SI directly influence individuals’ behavioral intention, whereas behavioral intention and FCs further influence actual use behavior; gender, age, experience, and voluntariness of use moderate these relationships. Specifically, PE refers to the extent to which an individual believes that using a new technology will improve work performance or bring benefits; EE refers to the perceived ease of using the technology; SI refers to the extent to which an individual perceives that important others or the social environment influence their use behavior; and FCs refer to the extent to which an individual perceives that organizational and technical infrastructures are available to support the use of the technology. At present, UTAUT has become one of the core theoretical models in technology adoption research [26] and has been widely used to explain and predict individuals’ intention to use and actual use of information technologies. Previous studies have shown that UTAUT has strong explanatory power for technology adoption intention and use behavior [27].

Given the high‐risk, high‐responsibility, and strongly bounded professional nature of nursing work, this study also treated perceived risk and professional boundaries as sensitizing concepts during interview design and analysis, in addition to the core constructs of UTAUT. However, these concepts were not treated as fixed final themes in advance; rather, their thematic formulation was further refined through analysis of the interview data. On this basis, UTAUT was used as the a priori analytical framework to explore nurses’ perceptions of LLMs, value judgments, intention to use, and related influencing factors.

#### 2.2.2. Development of the Interview Guide

Based on a review of relevant domestic and international literature and the aims of the study, the research team developed a semistructured interview guide. Guided by UTAUT and taking into account the high‐risk and high‐responsibility nature of nursing work, the guide also included questions designed to explore participants’ views on risk‐related concerns and professional boundaries. During the interviews, the researcher asked follow‐up questions as appropriate according to participants’ responses in order to obtain richer and more in‐depth data. The final interview guide included the following topics: (1) participants’ knowledge of LLMs, pathways of exposure, and experience of use; (2) their views on the value of LLMs in clinical nursing, teaching and training, research and learning, and nursing management; (3) their perceptions of the ease of use and convenience of LLMs; (4) the influence of social factors, such as colleagues, clinical preceptors, head nurses, and hospital attitudes, on their intention to use LLMs; (5) the influence of FCs, such as institutional regulations, technical support, and training guidance, on their intention to use LLMs; and (6) their views on the risks, professional boundaries, and conditions for future implementation of LLMs. Before formal data collection, the interview guide was pilot tested with two clinical nurses who met the inclusion criteria. After the pilot interviews, the research team discussed the clarity, relevance, and order of the questions and made minor revisions to improve the flow and comprehensibility of the interview guide. The pilot interviews were not included in the final analysis.

### 2.3. Data Collection

Data were collected through semistructured in‐depth interviews. Purposive sampling, guided by the principle of maximum variation, was used to recruit nurses with diverse characteristics, including hospital level, specialty, position, years of clinical experience, and educational background, in order to capture a broad range of perspectives and enhance data richness. Recruitment was facilitated through existing professional and educational/personal networks in order to access nurses from different hospital levels, specialties, and roles. This sampling strategy was intended to reflect potential differences in nurses’ perceptions, needs, and concerns regarding the use of LLMs across varying clinical and professional contexts. The interview guide was developed based on the study objectives and informed by the UTAUT framework. It was further refined through research team discussion before formal data collection.

Interviews were conducted by a researcher with a nursing background, prior training in qualitative interviewing, and experience in nursing research. The interviewer remained aware that professional familiarity with the topic might influence questioning and interpretation and therefore used open‐ended, nonleading prompts throughout the interviews. Some participants were known to the researchers through prior professional or educational contacts; however, there was no direct managerial, supervisory, or evaluative relationship between the interviewer and the participants during the study. Before each interview, the researcher explained the purpose of the study, the interview procedures, audio‐recording arrangements, and confidentiality principles to the participant. Formal interviews were conducted only after informed consent had been obtained.

All interviews were conducted in Chinese. Interviews were carried out in a combination of face‐to‐face and online formats, depending on participants’ availability and preference. The time and location of the interviews were determined in consultation with the participants, and a quiet, private, and distraction‐free environment was chosen whenever possible. Each interview lasted approximately 30–40 min. During the interviews, questions were asked flexibly based on the interview guide, and probing, clarification, and follow‐up prompts were used as appropriate to elicit richer and more in‐depth accounts. With participants’ permission, all interviews were audio‐recorded. At the same time, important nonverbal cues, such as tone, pauses, and emotional changes, were documented in field notes to preserve contextual information. Audio recordings were transcribed verbatim within 24 h after each interview and repeatedly checked against the recordings to ensure completeness and accuracy.

Data collection and analysis were conducted concurrently. After each interview, the transcript was reviewed and compared with previously coded data to assess whether new codes or themes were still emerging. The judgment of thematic saturation was made through ongoing discussion within the research team during the analytic process. The research team judged that thematic saturation was reached after the 15th interview, when no substantially new codes, categories, or themes were identified. To confirm the stability and completeness of the analytic framework, two additional interviews were conducted. These later interviews mainly served to verify the consistency of the existing themes rather than to generate new ones.

### 2.4. Data Analysis

Data were analyzed using directed content analysis [28]. The UTAUT served as the initial analytical framework. Specifically, the four core constructs of UTAUT—PE, EE, SI, and FCs—were used as preliminary coding categories to guide theory‐driven analysis of the interview data. In addition, risk‐related concerns and professional boundaries were treated as sensitizing concepts because they had been identified as relevant to nursing work during the design of the interview guide. However, they were not treated as predetermined final themes. During analysis, the research team remained open to inductive coding for data that could not be adequately captured by the original UTAUT framework. Through constant comparison and abstraction of the interview data, these materials were further refined into two complementary themes—perceived risk and professional boundaries and identity—which extended the explanatory scope of the original UTAUT framework in the context of nursing use of LLMs. The specific analytic steps were as follows: (1) the researchers repeatedly read the verbatim transcripts to obtain an overall understanding of the data; (2) meaning units relevant to the research topic, including words, sentences, and paragraphs, were identified; (3) these meaning units were initially coded and organized according to the UTAUT framework; (4) initial coding of the early transcripts was conducted independently by two researchers, after which the coding results were compared and areas of disagreement were discussed; (5) code definitions and category boundaries were refined through discussion, and content that could not be adequately classified within the original framework was subjected to open coding; (6) the revised coding framework was then applied to the remaining transcripts, with ongoing comparison across participants’ accounts; and (7) through constant comparison, repeated team discussion, and repeated reference to the original transcripts, the coding structure was further refined until the final themes were established. In this study, the first three transcripts were independently coded by two researchers. The coding results were then compared, and discrepancies in code definitions, category boundaries, and interpretation were resolved through discussion and repeated reference to the original transcripts until consensus was reached. After the initial coding framework had been refined, the remaining transcripts were coded by the first author and reviewed by another researcher. Coding decisions and theme development were discussed within the research team. Microsoft Excel was used to manage transcripts, codes, category matrices, coding memos, and theme development records.

### 2.5. Trustworthiness and Reflexivity

Reflexivity was maintained throughout the study because the researchers were themselves clinical nurses and nursing researchers. Their professional background facilitated communication with participants and supported a deeper understanding of the clinical context, but it also carried the potential risk of preconceptions during data collection and interpretation. To minimize this potential bias, the interviewers used a semistructured guide and open‐ended, nonleading questions, and the research team engaged in ongoing reflexive discussion throughout data collection and analysis. Interpretations were repeatedly checked against the original transcripts to ensure that themes were grounded in participants’ accounts rather than researchers’ prior assumptions.

To enhance trustworthiness, several strategies were used. First, two researchers independently coded the early transcripts, compared their coding results, and resolved discrepancies in code definitions, category boundaries, and interpretation through repeated discussion and reference to the original transcripts until consensus was reached. Second, field notes were used to preserve contextual information during data collection and interpretation. Third, data collection and analysis were conducted concurrently, allowing emerging themes to be continuously checked against subsequent interviews. These strategies helped strengthen the credibility and dependability of the findings and supported the transferability of the results to similar nursing contexts.

An audit trail was maintained throughout the analytic process, including original audio recordings, verbatim transcripts, field notes, coding memos, revisions to the coding framework, category matrices, and records of team discussions. During analysis, the research team also considered negative or divergent cases. Participant accounts that differed from the dominant patterns were compared with existing codes and categories and were used to refine theme boundaries and avoid overgeneralization of the findings.

All interviews were conducted and transcribed in Chinese. Quotations included in the manuscript were translated into English by the research team and checked against the original Chinese transcripts through team discussion in order to preserve participants’ intended meanings as accurately as possible.

### 2.6. Ethical Considerations

This study was approved by the Ethics Committee of the First Affiliated Hospital of Soochow University (approval no. 2025‐246), which served as the lead institution. The approved protocol covered the recruitment of eligible individual nurses through professional, educational, and personal networks. Although participants came from seven hospitals in Jiangsu and Shanghai, the other hospitals were not involved as formal participating research sites. Recruitment was not conducted through the institutional channels of these hospitals, and the study did not involve patient data, medical records, biological samples, clinical interventions, or institution‐level sensitive information from these hospitals. Participants from other hospitals took part voluntarily as individual professionals. Accordingly, no separate site‐level institutional permission or local ethics review was obtained from these hospitals. All participants provided informed consent before the interviews and were informed that participation was voluntary and that they could withdraw at any time. All procedures were conducted in accordance with the ethical principles outlined in the Declaration of Helsinki.

All audio recordings, transcripts, field notes, and coding documents were stored on password‐protected computers accessible only to the research team. Personal identifiers were removed during transcription and replaced with participant codes. Any information that could potentially identify participants, hospitals, colleagues, or patients was removed or generalized before analysis and reporting. The linkage file between participant identities and study codes was stored separately from the interview transcripts. Data will be retained in accordance with institutional and ethics requirements and then securely deleted or destroyed. For online interviews, participants were asked to join from a private environment to protect confidentiality. All participants provided informed consent for audio recording and for the use of anonymized quotations in publications.

This study was reported in accordance with the Standards for Reporting Qualitative Research (SRQR), and the completed SRQR checklist is provided in supporting file 1.

## 3. Results

Based on interview data from 17 nurses and analyzed using UTAUT‐guided directed content analysis, six themes were reported. Four themes corresponded to the predefined UTAUT analytical categories: PE, EE, SI, and FCs. Two additional themes, perceived risk and professional boundaries and identity, were refined from participants’ accounts related to the sensitizing concepts included in the interview guide. Participants expressed a positive but conditional intention to use LLMs. In this study, “conditional intention” is used as an interpretive finding derived from participants’ accounts, rather than as a validated new construct or a formal extension of UTAUT.

### 3.1. Theme 1: PE: Perceived Workload Reduction, Efficiency Gains, and Professional Support Were Described as Supporting Nurses’ Intention to Use LLMs

Most participants considered the most immediate appeal of LLMs to be their potential to reduce the burden of repetitive work, improve work efficiency, and provide support in areas such as knowledge assistance, teaching and training, research and learning, and nursing management. For nurses, the primary reason why LLMs are perceived as worth using is that they are useful.

#### 3.1.1. Perceived Reduction in the Burden of Documentation and Routine Tasks

The interviews showed that nurses generally viewed LLMs as supportive tools for reducing the burden of documentation and routine tasks. Across frontline nurses, clinical preceptors, and nursing administrators, participants consistently reported that nursing records, handover preparation, material writing, protocol revision, and report summarization consumed considerable time and energy, while LLMs were perceived as well suited to these standardized, repetitive, and text‐based tasks.

 P2: “AI can take over repetitive, tedious, and time‐consuming tasks such as writing records, searching for information, and organizing materials. In essence, it pulls nurses out of miscellaneous work so that they can focus on nursing itself.”
 P4: “Large language models can free us from those repetitive, mechanical, and time‐consuming tasks, so nurses can spend more time at the bedside, observing patients’ condition changes and communicating with family members. That is the core of nursing.”
 P6: “If AI could really handle those annoying records and forms, I would treasure it, because then I could spend more time checking on patients and talking with their families.” That is much better than sitting in front of a computer as a typist.”

#### 3.1.2. Perceived Support for Specialty Knowledge Access and Information Integration

In addition to reducing documentation burden, nurses generally expected LLMs to assist with specialty knowledge acquisition, compression of complex information, and navigation of difficult clinical issues. This was particularly evident in highly complex and uncertain settings such as the ICU, emergency department, NICU, and infectious diseases department, where participants especially valued the ability of LLMs to rapidly identify key points, summarize essential information, and reduce omissions within a short period of time.

 P3: “It is like a preceptor who is available 24 h a day. It can help me sort out key points for handover, standardize documentation, and adapt more quickly to the pace of ICU work.”
 P4: “When dealing with complex critically ill patients, it can help quickly integrate the key points and save time.”
 P14: “It is like my second brain.” It improves my efficiency in obtaining information and also helps me identify what I may have missed.”

#### 3.1.3. Perceived Support for Teaching and Training, Research and Learning, and Nursing Management

Support for teaching, research, and management was also a recurrent aspect of PE. Clinical preceptors, lead preceptors, postgraduate nurses, and nursing administrators generally believed that LLMs had strong potential in areas such as presentation preparation, question bank generation, design of ward round questions, scenario simulation, literature retrieval, research idea development, quality control data analysis, and administrative document drafting.

 P2: “I use it most often when writing papers, searching for literature, and organizing guidelines. I also ask AI to produce a first draft when writing departmental protocols, nursing routines, and training slides, which greatly improves efficiency.”
 P9: “LLMs respond very quickly when helping me complete quality control reports, official documents, and frontline problem‐solving, making our management more scientific and efficient.”
 P17: “In NICU nursing education, LLMs can help trainee nurses first build a knowledge framework, after which teachers can guide them to verify, supplement, and revise it.”

### 3.2. Theme 2: Effort Expectancy: Simplicity, User‐Friendliness, and Speed Were Described as Relevant to Continued Intention to Use LLMs

Although most participants acknowledged the value of LLMs, whether they were willing to continue using them in practice depended largely on whether the system was easy to use. During the interviews, words such as “simple,” “easy to use,” “fast,” and “not causing extra trouble” appeared repeatedly, indicating that EE potential influenced the transition from perceiving LLMs as useful to being willing to use them regularly.

#### 3.2.1. Perceived Ease of Operation as a Prerequisite for Sustained Intention to Use

Participants from different roles and age groups all emphasized that the system must be simple enough to use. Senior nurses, in particular, were highly sensitive to this issue, often expressing their expectations with phrases such as “I can’t deal with anything complicated,” “don’t make it too complex,” and “it should work with just a few clicks.” For them, learning a new technology itself constituted an additional burden; if the system was overly complex, it was difficult to develop a sustained intention to use it.

 P5: “AI must be fast, accurate, safe, and simple. It should not have complicated functions; otherwise, it just becomes a burden.”
 P11: “The system must be deeply integrated with existing nursing systems and must not create additional burden. Otherwise, in a high‐workload environment like the ICU, no one will want to use it, and even if they do, they will not feel comfortable relying on it.”
 P13: “I cannot handle complicated procedures; it should work with just a few clicks. Ideally, there should be no need to log in or figure things out.”

#### 3.2.2. Perceived Workflow Integration Enhances Usability

Many participants emphasized that LLMs should not remain isolated chat tools, but rather be truly embedded into existing workflows, with seamless connections to electronic medical records, nursing systems, monitoring devices, and workstations.

 P15: “If it cannot automatically retrieve patient information and fill in report forms and tables, then its value is limited.”
 P16: “If the system could automatically capture data from the monitors, or record what I say as I speak, that would greatly improve its practical value.”
 P17: “It needs to be embedded into the electronic medical record and nursing management systems so that it can be used smoothly without constantly switching between interfaces; otherwise, it will not truly become part of everyday NICU practice.”

#### 3.2.3. In High‐Pressure Settings, Speed and Output Format Were Perceived as Part of Usability

In the emergency department, ICU, and some high‐workload night‐shift situations, participants placed particular emphasis on system responsiveness and output format. If the system responded slowly, took too long to load, or produced lengthy and cumbersome outputs, it was considered not only inconvenient but also a potential risk in high‐pressure environments.

 P14: “In emergency care, it is not simply a question of whether AI is good or not; it is whether it fits the emergency setting and whether it can really help within a few seconds. If it takes dozens of seconds to generate an answer, or gives a long paragraph that I cannot immediately understand, it actually delays care.”
 P16: “If the system keeps loading, does not answer the question properly, or uses overly formal wording, I would not want to use it, because that kind of output is not practical and parents cannot understand it either.”

### 3.3. Theme 3: Social Influence: Peers, Preceptors, and the Organizational Climate Were Described as Relevant to Nurses’ Intention to Use LLMs

Nurses’ intention to use LLMs was not merely an individual attitude but was clearly influenced by peers, leaders, and organizational culture. Particularly in contexts where formal regulations have not yet been fully established, nurses tended to judge whether they could use LLMs, how they should use them, and whether continued use was worthwhile by observing the reactions of others.

#### 3.3.1. Peer Demonstration Facilitated Initial Exposure to and Experimentation With the Technology

Many participants reported that their first exposure to LLMs did not come from formal training, but rather from demonstrations by younger colleagues, trainee nurses, or friends around them.

 P4: “My first exposure to AI was when several nurses born after 1995 in our department were chatting during lunch break, saying that an AI tool could write nursing records. That was when I started to really try it.”
 P13: “It was only because the younger nurses in our department kept talking about it over the past two years that I gradually learned about it and occasionally watched how they used it.”
 P17: “I noticed that the nurses born after 2000 in our department are enthusiastic about using DeepSeek to make PowerPoint slides for nursing ward rounds, and the quality seems quite good. I may try it next time as well.”

#### 3.3.2. The Attitudes of Head Nurses, Clinical Preceptors, and Senior Nurses Influence Whether LLMs Are Used Openly

In addition to peer demonstration, the attitudes of head nurses, lead preceptors, and senior nurses exerted an even stronger influence on whether nurses felt confident enough to use LLMs openly, consistently, and appropriately. Many participants indicated that they felt more assured in trying the technology when leaders supported it and preceptors recognized its value and were willing to provide guidance.

 P4: “If my preceptor and head nurse both say that this tool is reliable, I would feel more comfortable using it.”
 P16: “If several senior nurses or the head nurse in the department took the lead in using it, everyone would definitely follow and learn.”

#### 3.3.3. Organizational Tolerance Coexists With Stigma of Use, Shaping the Spread of the Technology

A recurring pattern in the interviews was an organizational stance characterized by tacit tolerance without explicit encouragement. Many participants described the attitudes of hospitals and departments as “neither encouraging nor prohibiting,” “turning a blind eye,” or “allowing cautious exploration while remaining watchful.” This ambiguity provided nurses with some room to experiment but, at the same time, created a sense of stigma around use.

 P5: “At present, people mostly use it privately or during breaks.”
 P10: “The hospital’s current attitude toward AI is basically to turn a blind eye. Head nurses know that younger nurses are using it, but as long as it does not interfere with work or involve disclosure of patient information, they usually do not intervene too much.”
 P12: “The hospital repeatedly emphasizes patient privacy protection, medical safety, and documentation standards. The department allows cautious use in documentation, materials, and education, but everything must be checked by the user, direct copying is strictly prohibited, and no patient information may be shared externally.”

### 3.4. Theme 4: Facilitating Conditions: Organizational and Technical Safeguards Were Described as Supporting Cautious and Standardized Use of LLMs

The interviews showed that nurses were concerned not only with whether LLMs were useful or easy to use but also with the conditions under which they could use them with confidence, in a standardized manner, and over the long term.

#### 3.4.1. Intranet Security and Privacy Protection Were Regarded as Prerequisites for Use

Participants generally believed that, given the highly sensitive nature of patient privacy and clinical records, it would be difficult for LLMs to gain real trust if the system operated in an external network environment or allowed the direct uploading of medical records, behavioral notes, or risk‐related information.

 P15: “Information on patients with major infectious diseases is at the highest level of privacy. Ideally, the system should be deployed on the intranet and physically isolated from the external network.”
 P11: “The system must be intranet‐based only and should be reviewed by multidisciplinary experts in critical care, nursing, and infection control before it can be promoted in high‐risk departments.”

#### 3.4.2. Authoritative Traceability and Specialty Adaptation Were Perceived as Enhancing Trust

Many participants pointed out that nurses were not unwilling to accept AI; rather, they needed to know the basis on which a system generated its recommendations. Therefore, trust would be significantly enhanced if the system could directly provide sources such as clinical guidelines, textbooks, regulations, or internal institutional protocols. In high‐risk specialties, authoritative traceability was regarded as an important prerequisite for deciding whether to refer to AI‐generated suggestions.

 P5: “There must be a specialty‐specific version for the operating room. Do not try to fool us with a general version; we are completely different from wards or the ICU.”
 P17: “Knowledge from adult ICUs cannot simply be applied to neonatal care. The knowledge base must be reviewed and calibrated by pediatric and neonatal experts.”

#### 3.4.3. Workflow Integration, Clear Institutional Rules, and Training Support Were Seen as Enabling Practical Intention

Participants generally indicated that their intention to use LLMs depended in part on whether these tools could be integrated into existing systems, governed by clear institutional rules, and supported through adequate training, rather than functioning as an external tool that required constant switching between platforms. Many also suggested that clear rules should be established regarding what could and could not be done with LLMs, together with explicit accountability boundaries and usage standards, in order to reduce anxiety about being held responsible if something went wrong.

 P1: “Before implementation, the most important things are building trust, empowering staff through training, clarifying rules, and continuous optimization—especially helping nurses of different age groups develop safe and rational use capabilities.”
 P2: “Security and privacy must be strictly controlled; the answers must be accurate and professional; responsibility must be clearly defined; and the system must be simple to use and integrated with existing systems.”
 P11: “Formal implementation must address several core issues, including clear boundaries, explicit institutional rules, defined scenarios in which use is permitted or prohibited, clear accountability, and adequate tiered training—especially stronger guidance for younger nurses.”

### 3.5. Theme 5: Perceived Risk: Content Errors, Privacy Breaches, Unclear Accountability, and Erosion of Competence Were Perceived as Important Constraints on Intention to Use

Although most nurses acknowledged the potential value of LLMs, concerns about risk were repeatedly raised across almost all interviews. In the present study, perceived risk refers to nurses’ concerns about the potential adverse consequences of LLM use. Analysis of the 17 interviews showed several recurring high‐priority risks, including inaccurate content, privacy breaches, unclear accountability, erosion of competence, and homogenization of documentation. In addition, some participants raised more forward‐looking concerns, such as knowledge contamination and widening resource disparities.

#### 3.5.1. Inaccurate Content Was Perceived as the Most Central and Direct Source of Risk

For most participants, the first concern was whether LLMs might “sound convincing while actually being wrong.” This concern was particularly strong in high‐risk departments. For example, nurses in infectious diseases worried about incorrect recommendations regarding isolation and disinfection; psychiatric nurses worried about stigmatizing or provocative language triggering patient problems; neurology nurses worried about errors in assessing consciousness or swallowing that could delay treatment; and NICU nurses were particularly concerned about medication dosages, decimal points, and mismatches between AI output and individualized patient conditions.

 P13: “The content must be accurate. It cannot just talk nonsense. If you cannot tell whether it is right or wrong, then if something is wrong, it becomes a serious issue.”
 P16: “In the NICU, many drugs are dosed in micrograms per kilogram or milliliters. If the decimal point is off by one place, it becomes a major problem.”
 P12: “If the content generated by AI contains provocative language, it may trigger impulsive behavior, self‐harm, suicide, or aggression.” Therefore, in psychiatry, it cannot be allowed to cross the line into high‐risk communication and judgment.”

#### 3.5.2. Privacy Breaches and Unclear Accountability Were Perceived as Reducing the Sense of Safety in Use

Participants generally believed that medical records, behavioral documentation, infectious disease information, and data related to children were all highly sensitive and that any leakage could have serious consequences. At the same time, if an AI‐generated suggestion contributed to a nursing error, the responsibility might ultimately still fall on the nurse. As a result, many participants felt that although LLMs might be useful, they could not confidently rely on them.

 P8: “If a nursing error occurs because I referred to an AI‐generated suggestion, who would be held responsible?”
 P16: “If a problem arises because of an AI‐recommended medication dosage or nursing intervention, responsibility must be clearly defined.”

#### 3.5.3. Overreliance May Weaken Clinical Thinking

Erosion of competence was one of the most profession‐specific risks identified and was particularly emphasized by preceptors and senior nurses. They were generally concerned that if younger nurses came to treat AI as a “standard answer,” over time this might weaken bedside observation, active verification, independent judgment, and emergency response skills.

 P8: “AI can only play a supporting role, not the driving force.”
 P17: “What worries me most is that students stop thinking for themselves.” If they simply accept whatever AI says, how can clinical thinking be cultivated?
 P11: “Now, many younger nurses, when they encounter a problem, their first reaction is not to check the guidelines, not to think, and not to ask a senior nurse, but to look it up with AI directly. Over time, this will weaken their independent judgment, observational skills, and emergency response abilities.”
 P12: “What is most valuable in psychiatry is the ability to observe eye contact, facial expressions, tone of voice, and subtle movements. If those abilities are eroded by reliance on tools, it will directly affect the essence of nursing.”

In this theme, erosion of competence was treated primarily as a perceived consequence of overreliance on LLMs, that is, a risk associated with use, rather than as a normative statement about the legitimate role of AI in nursing practice.

#### 3.5.4. Homogenization of Documentation, Knowledge Contamination, and Technological Inequality Were Identified as Additional Concerns

Some participants also raised more systemic concerns. For example, nursing administrators worried that AI‐generated content might become overly templated, leading to nursing records that are overly uniform, with reduced individualization and diminished representation of patients’ dynamic conditions. There were also concerns about data contamination, whereby models might absorb nonstandard practices and then spread them in return. In addition, some participants expressed concern that if high‐quality AI resources become concentrated in large hospitals, disparities in nursing resources may be further widened.

 P17: “If high‐quality AI resources are concentrated in large hospitals in the future, this may further widen the gap in nursing resources.”

### 3.6. Theme 6: Professional Boundaries and Identity: Nurses’ Intention to Use AI Is Conditional

Although nurses generally acknowledged the supportive value of LLMs, they also demonstrated a very clear awareness of role boundaries. Unlike Theme 5, which focuses on the anticipated harms associated with LLM use, this theme addresses nurses’ normative judgments about what AI should not do in nursing practice and why certain aspects of care must remain under human professional control. What participants accepted was not the full‐scale entry of AI into nursing, but rather AI participation in nursing only on the condition that it did not cross professional boundaries.

#### 3.6.1. AI Can Only Serve as a Supportive Tool; It Cannot Take the Lead, and It Certainly Cannot Be a Teacher

Different participants repeatedly expressed this point in different ways. Taken together, these statements indicate that nurses are willing to accept a tool that can provide assistance, build frameworks, and help organize thinking, but not a system that can replace judgment or dominate practice.

 P2: “It can be an assistant, but it must never be the main actor.”
 P5: “AI can only help; it cannot take the lead. It can only be used for reference, not relied on. It can only improve speed; it cannot take responsibility for me.”
 P17: “It can be a tool, but it cannot be a teacher; it can support decision‐making, but it cannot replace thinking.”

#### 3.6.2. High‐Risk Clinical Decision‐Making Scenarios Are Clear Red Lines

Participants across all specialties were able to clearly define the boundaries of AI application. They generally accepted its use in standardized, repetitive, low‐risk, and text‐based tasks but explicitly rejected its use in scenarios involving condition assessment, clinical decision‐making, emergency response, risk assessment, and emotionally charged communication.

 P11: “Any stage involving patient safety, clinical judgment, nursing decision‐making, emergency response, condition assessment, treatment coordination, procedural guidance, medication monitoring, or risk assessment must strictly prohibit the use of AI.”
 P12: “AI can only be a writing assistant, an information assistant, or a standardization assistant; it must never become a judgment assistant, a decision‐making assistant, or an intervention assistant.”
 P16: “It is an assistant, not the boss; it can help with tasks, but it cannot be the brain.”

#### 3.6.3. The Human Warmth of Nursing, Empathy, and Clinical Insight Are Irreplaceable

In the interviews, nurses defined the boundaries of AI not only from the perspective of safety but also from the perspective of the essence of nursing, emphasizing that human warmth, empathic support, and clinical insight cannot be replaced by technology.

 P9: “AI can change the efficiency of nursing, but it can never change the essence of nursing, because it cannot replace nurses’ ability to observe, communicate, empathize, and convey warmth.”
 P8: “No matter how advanced the technology is, it cannot replace nurses’ eyes in observing a patient’s condition, nor can it replace the warmth of a nurse holding a patient’s hand.”
 P17: “Technology is cold, but our hands must remain warm.”

Overall, the findings suggest that nurses’ intention to use LLMs was characterized not by unconditional intention or outright resistance but by a pattern of conditional intention. Participants recognized the practical value of LLMs in improving efficiency and supporting routine tasks, yet their intention to use these tools remained constrained by concerns about safety, accountability, and the boundaries of nursing practice. Nurses’ intention of LLMs, therefore, appeared to involve a continuous balancing of expected benefits against professional, ethical, and contextual considerations.

## 4. Discussion

### 4.1. Clinical Nurses Generally Showed a Positive Yet Conditional Intention to Use LLMs

This study found that nurses generally held positive or cautiously positive attitudes toward LLMs; however, such intention was clearly conditional rather than unconditional. Participants recognized the perceived practical value of LLMs in documentation, teaching, research, management, and knowledge support, indicating that these technologies are no longer perceived as entirely unfamiliar or unacceptable in nursing contexts. This overall pattern is broadly consistent with adjacent literature showing that nurses and other healthcare professionals often approach AI with guarded optimism, while emphasizing that it should support rather than replace human judgment, empathy, and professional responsibility [13, 29]. At the same time, the present findings suggest that nurses did not evaluate LLMs in the same way that users might evaluate general consumer technologies. Instead, their intention to use LLMs reflected a process of conditional intention, in which expected usefulness was continuously weighed against patient safety, professional accountability, organizational legitimacy, and the boundaries of nursing practice. In this sense, the study extends adjacent nursing AI literature by providing a more context‐specific account of how clinical nurses, as the direct users of LLMs in real‐world nursing settings, negotiate their engagement with these tools. Whereas previous studies have highlighted attitudes, readiness, facilitators and barriers, and professional role‐related concerns in relation to AI [30], the present study further shows that clinical nurses’ engagement with LLMs is better understood not as simple acceptance or rejection but as a form of conditional intention shaped by perceived risk, professional boundaries, and professional identity. This interpretation also resonates with broader literature suggesting that AI integration in professional practice is closely tied to questions of professional self, autonomy, and identity negotiation and that AI is often accepted as a complementary aid rather than as a substitute for professional agency [31, 32]. Taken together, these findings suggest that the adoption of LLMs in nursing should be understood as a profession‐specific form of technology intention shaped by the high‐risk, high‐responsibility, and human‐centered nature of nursing work.

### 4.2. Mechanisms Underlying Nurses’ Intention to Use LLMs Within the UTAUT Framework

The findings not only suggest that UTAUT provides a useful framework for understanding nurses’ intention to use LLMs but also indicate that these mechanisms are reinterpreted through the specific logic of nursing work. In the present study, the four UTAUT‐related constructs did not operate as isolated predictors. Rather, they formed a linked process through which nurses evaluated whether LLMs were worth using, manageable in practice, professionally legitimate, and organizationally safe.

First, PE was the most immediate basis for positive intention to use, but its meaning in nursing extended beyond general perceptions of usefulness. Participants valued LLMs primarily when they could reduce repetitive documentation, support information retrieval, and improve efficiency in teaching, research, and management, thereby releasing time and attention for direct patient care and other higher value professional activities [33]. This suggests that perceived usefulness in nursing is not innovation‐oriented in an abstract sense, but strongly task‐dependent and grounded in the practical realities of clinical work. In other words, nurses were not attracted to LLMs because they represented technological advancement per se, but because they appeared capable of alleviating low‐value workload and returning attention to the bedside.

Second, EE may influence whether this perceived usefulness could be translated into sustained intention to use. In the nursing context, usability was not limited to technical simplicity; rather, it was closely tied to response speed, concise output, low cognitive burden, and smooth fit with existing workflows, especially in high‐pressure settings [34]. Thus, EE in this study is better understood as contextual fit than as ease of operation alone. A system that is theoretically useful may still fail to be adopted if it disrupts workflow, demands extra effort, or cannot support timely action in busy clinical environments. This finding indicates that, in nursing, usefulness is filtered through the realities of workload, pace, and workflow integration.

Third, SI shaped not only whether participants first encountered LLMs but also whether they perceived their use as professionally legitimate. Peer demonstration lowered the threshold for initial experimentation, whereas endorsement from preceptors, head nurses, and senior staff increased confidence that LLM use was acceptable and professionally defensible. Conversely, ambiguous organizational attitudes reinforced caution and stigma, limiting visible or sustained use even when nurses recognized the value of the technology [35]. These findings suggest that SI in nursing is not merely a matter of interpersonal persuasion. Rather, it functions as a signal of legitimacy within a highly organized professional environment in which nurses continuously assess whether a new technology is institutionally tolerated, professionally endorsed, and socially safe to use.

Finally, FCs may influence whether intention to use could be translated into practical adoption. Participants described intranet security, authoritative traceability, specialty adaptation, workflow integration, and training not simply as desirable technical features but as organizational safeguards that made LLM use feel trustworthy, governable, and professionally acceptable [9, 36]. In this sense, FCs in nursing are not neutral implementation supports; they are closely tied to governance, role clarity, and psychological safety. Nurses were more willing to adopt LLMs when organizational and technical arrangements reduced uncertainty, clarified accountability, and reinforced the legitimacy of using these tools in practice.

Taken together, these findings suggest that intention to use LLMs in nursing is not shaped by perceived usefulness alone but by a staged and context‐sensitive appraisal of utility, usability, legitimacy, and safety. This also explains why UTAUT is helpful but not sufficient on its own in the nursing context: While it captures the enabling side of technology intention, its constructs in practice are deeply shaped by the high‐risk, high‐responsibility, and strongly bounded nature of nursing work.

### 4.3. Factors Influencing Nurses’ Intention to Use LLMs in the Nursing Context

#### 4.3.1. Perceived Risk Was Described as an Important Source of Caution Regarding LLM Use

A major contribution of this study is the finding that perceived risk emerged as an important constraint on nurses’ intention to use LLMs and represents an important dimension not fully captured by the original UTAUT framework. Participants’ concerns extended beyond abstract uncertainty about technology to concrete fears of inaccurate content, privacy breaches, unclear accountability, and the erosion of younger nurses’ clinical competence [34]. Importantly, these concerns were closely tied to the safety‐sensitive and responsibility‐laden nature of nursing work. When the potential consequences of error involved patient harm, professional liability, or weakened clinical reasoning, perceived risk often outweighed perceived usefulness. This finding suggests that in high‐stakes care environments, technology intention cannot be adequately explained without considering how users anticipate and interpret risk [37].

#### 4.3.2. Professional Boundaries and Identity Were Reflected in Nurses’ Accounts of Conditional Intention to Use LLMs

Another particularly nursing‐specific finding was that professional boundaries and identity profoundly shaped nurses’ conditional intention to use LLMs. Although this theme is related to perceived risk, the two are analytically distinct. Perceived risk concerns nurses’ anticipation of adverse consequences from LLM use, whereas professional boundaries and identity concern nurses’ judgments about the legitimate role of AI in relation to professional responsibility, clinical judgment, and the human‐centered essence of nursing. Participants consistently described AI as an “assistant,” “helper,” or “tool” but rejected its positioning as a “teacher,” “decision‐maker,” or substitute for professional judgment. This boundary awareness was especially evident in situations involving condition assessment, emergency response, medication‐related judgment, crisis communication, and humanistic support. These findings indicate that nurses did not assess LLMs solely in terms of efficiency or functionality; rather, they evaluated them through the lens of professional responsibility, relational care, and the human‐centered essence of nursing [38]. Therefore, nurses’ intention to use LLMs is better understood as a form of bounded or conditional intention, insofar as such use remains subordinate to human judgment, empathy, and accountability.

Taken together, these findings suggest that UTAUT provides a useful but incomplete framework for understanding nurses’ intention to use LLMs. While PE, EE, SI, and FCs explained important enabling mechanisms, perceived risk and professional boundaries emerged as complementary dimensions that extended the explanatory scope of the model in the nursing context. The concept of conditional intention may, therefore, offer a more precise interpretation of how nurses engage with LLMs in high‐risk and human‐centered care environments.

### 4.4. Implications for Nursing Management

The findings suggest several priorities for cautious pilot testing of LLM‐supported nursing applications rather than immediate large‐scale implementation. Nurse managers may consider starting with low‐risk and clearly bounded scenarios, such as documentation support, health education material preparation, teaching and training support, and general knowledge retrieval. For scenarios involving nursing diagnosis, care planning, or clinical decision‐making, LLM outputs should be treated only as auxiliary references and should remain subject to professional verification and accountability.

Before implementation, nursing managers should establish clear boundaries for appropriate and inappropriate use, including rules on patient privacy, data input, documentation, human review, and responsibility attribution. Training should emphasize not only operational skills but also risk awareness, information verification, ethical use, and the limits of LLM‐generated content. Pilot testing should be accompanied by continuous monitoring of usability, workflow fit, perceived workload, documentation quality, patient‐safety concerns, and nurses’ feedback. These steps may help healthcare organizations explore the potential role of LLMs in nursing while avoiding premature assumptions about their effectiveness or safety.

### 4.5. Limitations

This study has several limitations. First, although participants were purposefully selected to include nurses from different hospital levels, specialties, and roles, they were still drawn from a limited number of regions and institutions, which may restrict the transferability of the findings. In particular, the perspectives represented in this study may more strongly reflect nurses working in relatively resource‐rich settings. Second, one of the inclusion criteria required participants to have at least some knowledge of or experience using LLMs. As a result, nurses with very low awareness, no prior exposure, or strongly skeptical views toward LLMs may have been underrepresented in the sample. This may have narrowed the range of perspectives captured in the study, particularly those of non‐users or nurses who remain resistant to AI adoption. Third, this was a qualitative study based primarily on interview data and focused on intention to use rather than actual use behavior or implementation outcomes. Fourth, because LLM technologies are evolving rapidly, nurses’ attitudes and expectations may change over time as institutional policies, system capabilities, and practical experience develop. In addition, related person‐centered research has suggested meaningful heterogeneity in nurses’ attitudes toward artificial intelligence at work, indicating that “conditional intention” may not be uniform across subgroups of nurses. Future studies could employ larger‐scale quantitative or mixed‐methods designs to further test the factors and thematic relationships identified in this study, include nurses with little or no prior exposure to LLMs, combine latent profile analysis with qualitative inquiry to explore potential gradations in intention, and examine specialty‐specific implementation models and governance strategies for LLM use in nursing.

## 5. Conclusion

Guided by UTAUT, this qualitative study explored the intention to use LLMs among 17 clinical nurses with prior familiarity with or experience using these tools. The findings suggest that participants expressed a positive but conditional intention to use LLMs. Their intention was reflected in accounts of perceived usefulness, ease of use, SI, FCs, perceived risk, and professional boundaries. Participants recognized the potential supportive value of LLMs particularly in standardized, supportive, and relatively low‐risk tasks, while remaining cautious about their use in high‐risk scenarios involve clinical judgment, emergency response, individualized patient care, and humanistic communication. Overall, “conditional intention” should be understood as an interpretive finding derived from participants’ accounts, rather than as a validated new construct or a formal extension of UTAUT. The findings should, therefore, be interpreted as context‐specific insights from nurses with prior LLM familiarity or use, rather than as evidence of general acceptance among all clinical nurses.

## Author Contributions

Xu Li: conceptualization, data curation, formal analysis, funding acquisition, investigation, methodology, visualization, writing–original draft, and writing–review and editing; Huiting Xu and Xu Hu: data curation, formal analysis, methodology, visualization, and writing–review and editing; Jingjing Guo: data curation and writing–review and editing; Pin Yu and Hailing Ju: conceptualization, methodology, validation, writing–review and editing, supervision, project administration, and funding acquisition.

## Funding

This study was supported by the “Zhou” Nursing Research Project of the First Affiliated Hospital of Soochow University (project no. HLYJ‐Z‐202502) and the 2026 Hospital Management Innovation Research Project of the Jiangsu Provincial Hospital Association (project no. JSYGY‐3‐2026‐125).

## Disclosure

The funders had no role in the study design, data collection, data analysis, interpretation of the findings, manuscript preparation, or decision to submit the manuscript for publication.

## Ethics Statement

This study was approved by the Ethics Committee of the First Affiliated Hospital of Soochow University (approval no. 2025‐246). All participants provided informed consent and participated voluntarily. All procedures were conducted in accordance with the ethical principles outlined in the Declaration of Helsinki.

## Conflicts of Interest

The authors declare no conflicts of interest.

## Supporting Information

Additional supporting information can be found online in the Supporting Information section.

## Supporting information


**Supporting Information** Supporting File 1. SRQR Checklist. Completed Standards for Reporting Qualitative Research (SRQR) checklist for this study.

## Data Availability

The full interview transcripts are not publicly available because they contain information that could compromise participant confidentiality. The data underlying this article may be shared upon reasonable request from the corresponding author.

## References

[1] Buchan J. , Catton H. , and Shaffer F. , Ageing Well? Policies to Support Older Nurses at Work, International Centre on Nurse Migration. (2020) 1–48.

[2] ICN , The Global Nursing Shortage and Nurse Retention, 2021, International Council of Nurses, Geneva, Switzerland.

[3] Li H. Q. , Xie P. , Huang X. , and Luo S. X. , The Experience of Nurses to Reduce Implicit Rationing of Nursing Care: A Phenomenological Study, BMC Nursing. (2023) 22, no. 1, 10.1186/s12912-023-01334-5. PMC10197034 37208756

[4] Stevens E. R. , Alfaro Arias V. , Luu S. , Lawrence K. , and Groom L. , Technology Integration to Support Nurses in An “Inpatient Room of the Future”: Qualitative Analysis, Journal of Medical Internet Research. (2025) 27, 10.2196/68689. PMC12209724 40522717

[5] Iqbal U. , Tanweer A. , Rahmanti A. R. , Greenfield D. , Lee L. T. , and Li Y. J. , Impact of Large Language Model (ChatGPT) in Healthcare: An Umbrella Review and Evidence Synthesis, Journal of Biomedical Sciences. (2025) 32, no. 1, 10.1186/s12929-025-01131-z. PMC12057020 40335969

[6] Bednarczyk L. , Reichenpfader D. , Gaudet-Blavignac C. et al., Scientific Evidence for Clinical Text Summarization Using Large Language Models: Scoping Review, Journal of Medical Internet Research. (2025) 27, 10.2196/68998. PMC12123242 40371947

[7] Yu H. , Zhou J. , Li L. et al., Simulated Patient Systems Powered by Large Language Model-Based AI Agents Offer Potential for Transforming Medical Education, Communication and Medicine. (2025) 6, no. 1, 10.1038/s43856-025-01283-x. PMC12808140 41420084

[8] O’Sullivan J. W. , Palepu A. , Saab K. et al., A Large Language Model for Complex Cardiology Care, Nature Medicine. (2026) 32, no. 2, 616–623, 10.1038/s41591-025-04190-9. PMC12920087 41652123

[9] Hobensack M. , von Gerich H. , Vyas P. et al., A Rapid Review on Current and Potential Uses of Large Language Models in Nursing, International Journal of Nursing Studies. (2024) 154, 10.1016/j.ijnurstu.2024.104753. 38560958

[10] El Arab R. A. , Al Moosa O. A. , Abuadas F. H. , and Somerville J. , The Role of AI in Nursing Education and Practice: Umbrella Review, Journal of Medical Internet Research. (2025) 27, 10.2196/69881. PMC12008698 40072926

[11] Woo B. F. Y. , Cato K. , Cho H. , You S. B. , and Song J. , The Use of Large Language Models in Clinical Documentation: A Scoping Review, International Journal of Nursing Studies. (2026) 176, 10.1016/j.ijnurstu.2025.105322. 41512731

[12] Pare G. , Raymond L. , and Etindele Sosso F. A. , Nurses’ Intention to Integrate AI Into Their Practice: Survey Study in Canada, JMIR Nursing. (2025) 8, 10.2196/76795. PMC12413143 40911813

[13] Ramadan O. M. E. , Alruwaili M. M. , Alruwaili A. N. , Elsehrawy M. G. , and Alanazi S. , Facilitators and Barriers to AI Adoption in Nursing Practice: A Qualitative Study of Registered Nurses’ Perspectives, BMC Nursing. (2024) 23, no. 1, 10.1186/s12912-024-02571-y. PMC11654280 39695581

[14] Fan J. , Zhang L. , Li N. , and Man S. , How the Perceived Substitution of AI Technology Hinders Nurses’ Innovation Behavior: The Mediating Role of AI Anxiety and Human-AI Cooperation Intention and the Moderating Role of Organizational AI Readiness, BMC Nursing. (2025) 24, no. 1, 10.1186/s12912-025-03265-9. PMC12224676 40611103

[15] Hassan M. , Kushniruk A. , and Borycki E. , Barriers to and Facilitators of Artificial Intelligence Adoption in Health Care: Scoping Review, JMIR Human Factors. (2024) 11, 10.2196/48633. PMC11393514 39207831

[16] Zhao Y. , Yuan Y. , Wen Z. et al., The Current Status, Knowledge, Attitudes, and Challenges of Generative Artificial Intelligence Use Among Undergraduate Nursing Students: A Single-Center Cross-Sectional Survey of Western China, Frontiers in Public Health. (2025) 13, 10.3389/fpubh.2025.1648416. PMC12479433 41036128

[17] BinJwair A. and Alamer A. , Digital Drivers: How the Acceptance and Use of Technology Model Shapes University Students’ Behavior Toward Generative AI, Acta Psychologica. (2025) 261, 10.1016/j.actpsy.2025.105930. 41274011

[18] Alenazi L. and Alhalal E. , Factors Affecting Artificial Intelligence Usage Intention Among Nursing Students: Unified Theory of Acceptance and Use of Technology, Nurse Education Today. (2025) 152, 10.1016/j.nedt.2025.106780. 40382862

[19] Mohamed M. G. , Goktas P. , Khalaf S. A. et al., Generative Artificial Intelligence Acceptance, Anxiety, and Behavioral Intention in the Middle East: A TAM-Based Structural Equation Modelling Approach, BMC Nursing. (2025) 24, no. 1, 10.1186/s12912-025-03436-8. PMC12211728 40598322

[20] Jiang H. , Wang Z. , Meng M. et al., Cognitive Status of Nursing Postgraduates Toward Generative Artificial Intelligence: A Qualitative Study Based on the UTAUT Framework, BMC Nursing. (2026) 25, no. 1, 10.1186/s12912-025-04187-2. PMC12869936 41501802

[21] Siah C. R. , Lim J. V. , Lyu M. M. , Levett-Jones T. , and Mattar I. , Authenticity and Academic Integrity in Generative Artificial Intelligence (GenAI) Use Among Undergraduate Nursing Students, Journal of Professional Nursing. (2026) 62, 45–51, 10.1016/j.profnurs.2025.10.007. 41679804

[22] Shen M. , Shen Y. , and Shi Z. , Exploring Nursing Students’ Acceptance of RAG-Enhanced GenAI Through the AIDUA Model: A Qualitative Study, Nurse Education in Practice. (2025) 89, 10.1016/j.nepr.2025.104612. 41161072

[23] Guo Y. , Guo J. , Qiu Y. et al., A Qualitative Study on the Willingness and Application Demands of Clinical Nurses in the Use of Large Language Models, Research Square. (January 2026) 10.21203/rs.3.rs-8471430/v1.

[24] Takona J. P. , Research Design: Qualitative, Quantitative, and Mixed Methods Approaches/Sixth Edition, Quality and Quantity. (2024) 58, no. 1, 1011–1013, 10.1007/s11135-023-01798-2.

[25] Venkatesh V. , Morris M. G. , Davis G. B. , and Davis F. D. , User Acceptance of Information Technology: Toward a Unified View, MIS Quarterly. (2003) 27, no. 3, 425–478, 10.2307/30036540.

[26] Wutz M. , Hermes M. , Winter V. , and Koberlein-Neu J. , Factors Influencing the Acceptability, Acceptance, and Adoption of Conversational Agents in Health Care: Integrative Review, Journal of Medical Internet Research. (2023) 25, 10.2196/46548. PMC10565637 37751279

[27] Zhao J. and Wang J. F. , Health Advertising on Short-Video Social Media: A Study on User Attitudes Based on the Extended Technology Acceptance Model, International Journal of Environmental Research and Public Health. (2020) 17, no. 5, 10.3390/ijerph17051501. PMC7084731 32110950

[28] Hsieh H. F. and Shannon S. E. , Three Approaches to Qualitative Content Analysis, Qualitative Health Research. (2005) 15, no. 9, 1277–1288, 10.1177/1049732305276687. 16204405

[29] El Arab R. A. , Alshakihs A. H. , Alabdulwahab S. H. et al., Artificial Intelligence in Nursing: A Systematic Review of Attitudes, Literacy, Readiness, and Adoption Intentions Among Nursing Students and Practicing Nurses, Frontiers in Digital Health. (2025) 7, 10.3389/fdgth.2025.1666005. PMC12507812 41079691

[30] Ta’an W. , Damrah S. , Al-Hammouri M. M. , and Williams B. , Professional Identity and Its Relationships With AI Readiness and Interprofessional Collaboration, PLoS One. (2025) 20, no. 5, 10.1371/journal.pone.0322794. PMC12083793 40378129

[31] Schneider-Kamp A. and Godono A. , The AI-Extended Professional Self: User-Centric AI Integration Into Professional Practice With Exemplars From Healthcare, AI & Society. (2025) 40, no. 7, 5469–5480, 10.1007/s00146-025-02319-5.

[32] Terlinden L. , Verachtert A. , and Bollens J. , AI in Healthcare: Identity Threat or Opportunity? Insights From Medical Specialists, Qualitative Health Research. (2026) 36, no. 2/3, 289–303, 10.1177/10497323251387568. 41255039

[33] Mikkonen K. , Tuunainen S. , Oikarinen A. et al., Artificial Intelligence Technologies Supporting Nurses’ Clinical Decision-Making: A Systematic Review, Journal of Clinical Nursing. (2026) 35, no. 4, 1525–1540, 10.1111/jocn.70156. 41292265 PMC12964510

[34] Lambert S. I. , Madi M. , Sopka S. et al., An Integrative Review on the Acceptance of Artificial Intelligence Among Healthcare Professionals in Hospitals, NPJ Digital Medicine. (2023) 6, no. 1, 10.1038/s41746-023-00852-5. PMC10257646 37301946

[35] Kotp M. H. , Ismail H. A. , Basyouny H. A. A. et al., Empowering Nurse Leaders: Readiness for AI Integration and the Perceived Benefits of Predictive Analytics, BMC Nursing. (2025) 24, no. 1, 10.1186/s12912-024-02653-x. PMC11737245 39819624

[36] Burford J. S. , Booth R. G. , and McIntyre A. , Exploring the Intersection of Nursing Leadership and Artificial Intelligence: Scoping Review, JMIR Nursing. (2025) 8, 10.2196/80085. PMC12617831 41237291

[37] Joo J. Y. , Liu M. F. , and Ho M. H. , Nurses’ Perceptions of Artificial Intelligence Adoption in Healthcare: A Qualitative Systematic Review, Nurse Education in Practice. (2025) 88, 10.1016/j.nepr.2025.104542. 40925173

[38] Hassan E. A. and El-Ashry A. M. , Leading With AI in Critical Care Nursing: Challenges, Opportunities, and the Human Factor, BMC Nursing. (2024) 23, no. 1, 10.1186/s12912-024-02363-4. PMC11475860 39402609

